# Supplementary material for: A Terpyridine-Fe2+-Based Coordination Polymer Film for On-Chip Micro-Supercapacitor with AC Line-Filtering Performance
Source: Polymers (Basel). 2021 Mar 24;13(7):1002. doi: 10.3390/polym13071002 (PMC8037160; doi:10.3390/polym13071002)
Supplement: Supplementary file 1 [file polymers-13-01002-s001.pdf]

polymers

Supporting Information

A Terpyridine-Fe^{2+}-Based Coordination Polymer Film for On-Chip Micro-Supercapacitor with AC Line-Filtering Performance

Hongxing Wang [1], Feng Qiu [1,*], Chenbao Lu [2], Jinhui Zhu [2], Changchun Ke [3], Sheng Han [1,*] and Xiaodong Zhuang [2,*]

1 School of Chemical and Environmental Engineering, Shanghai Institute of Technology, Haiquan Road 100, Shanghai 201418, China; wang_hx1910@163.com
2 The meso-Entropy Matter Lab, The State Key Laboratory of Metal Matrix Composites, Shanghai Key Laboratory of Electrical Insulation and Thermal Ageing, School of Chemistry and Chemical Engineering, Frontiers Science Center for Transformative Molecules, Shanghai Jiao Tong University, Shanghai 200240, China; castle@sjtu.edu.cn (C.L.); zhujinhui1109@sjtu.edu.cn (J.Z.)
3 School of Mechanical Engineering, Shanghai Jiao Tong University, 200240, Shanghai, China; kechangchun@sjtu.edu.cn
* Correspondence: fengqiu@sit.edu.cn (F.Q.); hansheng654321@sina.com (S.H.); zhuang@sjtu.edu.cn (X.Z.)

Citation: Wang, H.; Qiu, F.; Lu, C.; Zhu, J.; Ke, C.; Han, S.; Zhuang, X. A Terpyridine-Fe^{2+}-Based Coordination Polymer Film for On-Chip Micro-Supercapacitor with AC Line-Filtering Performance. *Polymers* **2021**, *13*, 1002. https://doi.org/10.3390/polym13071002

Academic Editor: Chen-I Yang

Received: 9 March 2021
Accepted: 22 March 2021
Published: 24 March 2021

Publisher's Note: MDPI stays neutral with regard to jurisdictional claims in published maps and institutional affiliations.

Copyright: © 2021 by the authors. Licensee MDPI, Basel, Switzerland. This article is an open access article distributed under the terms and conditions of the Creative Commons Attribution (CC BY) license (http://creativecommons.org/licenses/by/4.0/).

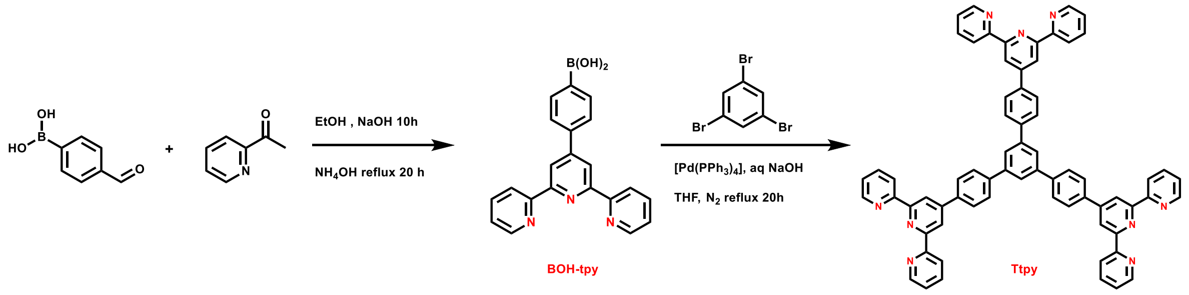

Scheme S1. Synthesis route to TpPB.

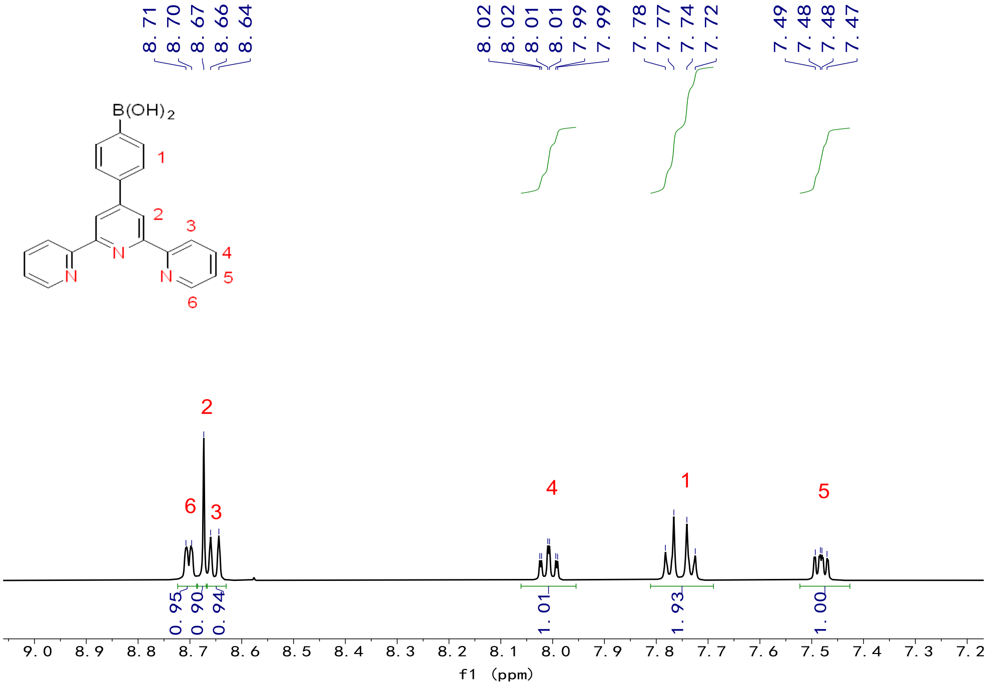

Figure S1. The ^{1}H NMR spectrum of 4′-(2,2′:6′,2′′-terpyridine)phenylboracic acid.

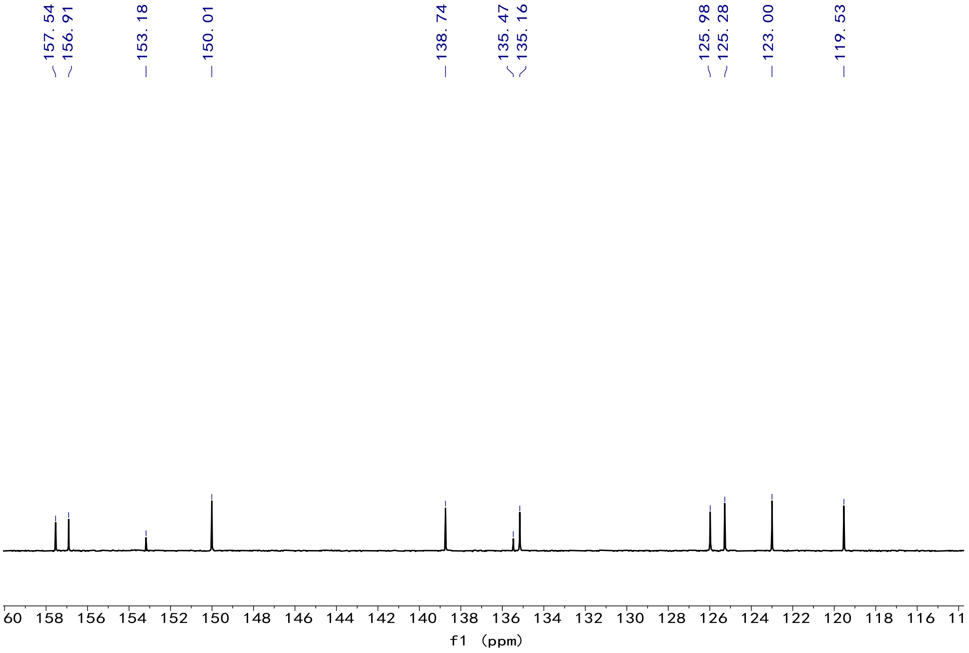

Figure S2. The ^{13}C NMR spectrum of 4′-(2,2′:6′,2′′-terpyridine)phenylboracic acid.

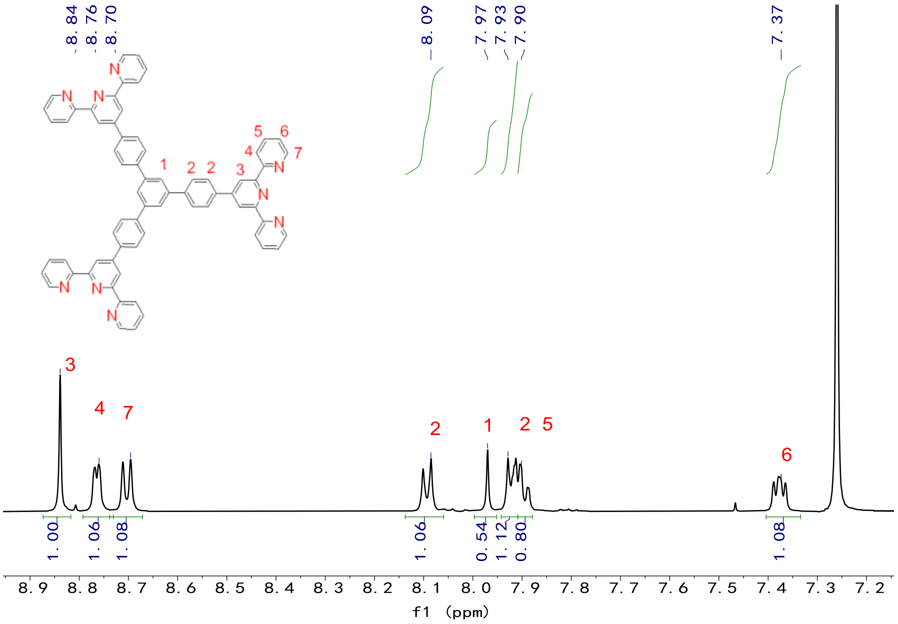

Figure S3. The ^{1}H NMR spectrum of 1,3,5-tri(4-(2,2′:6′,2″-terpyridine)phenyl) benzene.

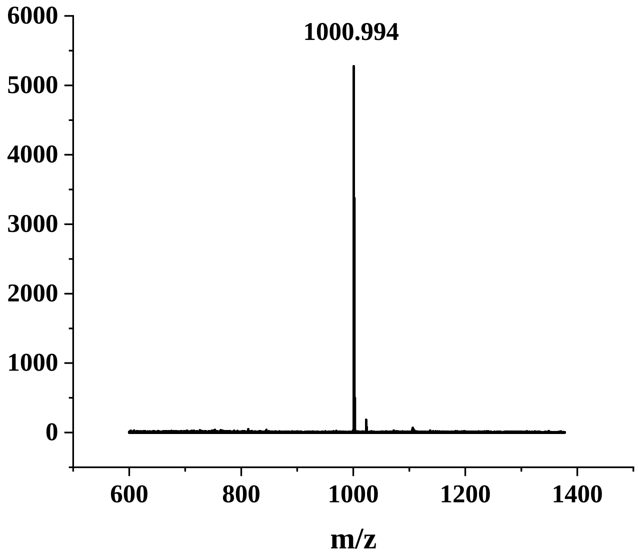

Figure S4. The MALDI-TOF of 1,3,5-tri(4-(2,2′:6′,2″-terpyridine)phenyl)benzene.

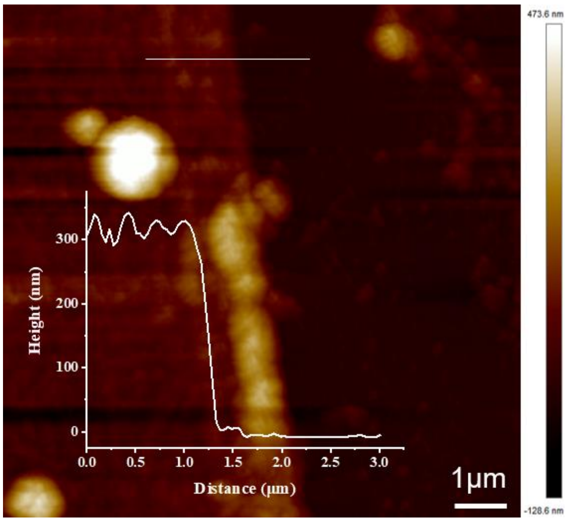

Figure S5. AFM image of **TpPB-Fe** film.

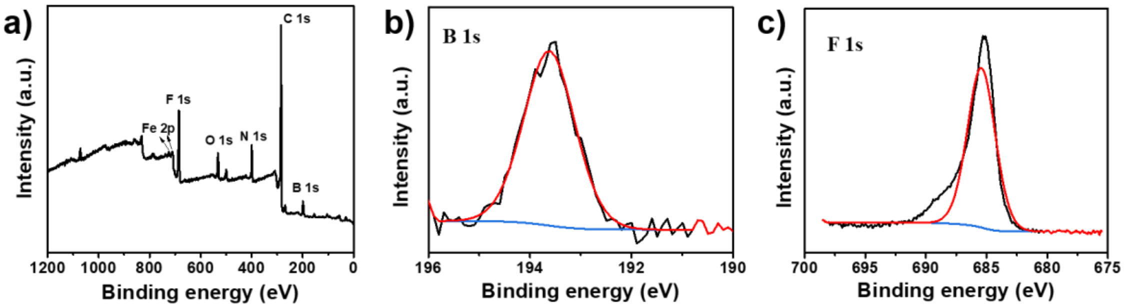

Figure S6. **a**) XPS survey spectrum, **b**) B 1s XPS spectrum, **c**) F 1s XPS spectrum of TpPB-Fe.

a)

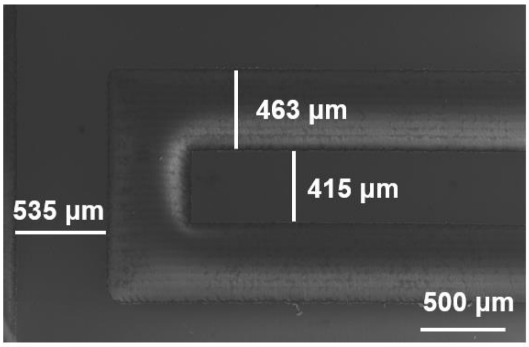

b)

Interdigital Finger Number, N	12
Finger Width, μm	415
Finger Length, mm	7
Interspatial Distance, μm	463
Edge Breath, μm	535
Effective Area, cm^2	0.45

$$0.415*7*12+0.535*(0.415+0.463)*10+0.415)*2=44.698\ mm^2$$

Figure S7. The parameters of the prepared Au interdigitated electrodes.

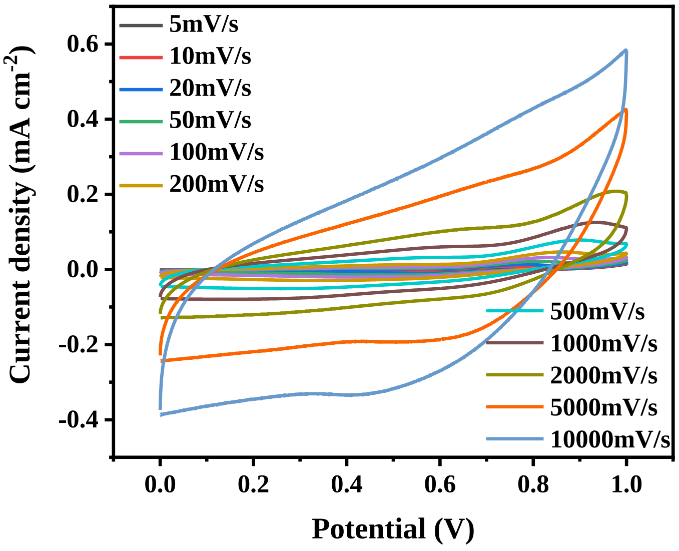

Figure S8. CV curves of TpPB-Fe-based MSC in PVA/LiCl gel electrolyte at different scan rates.

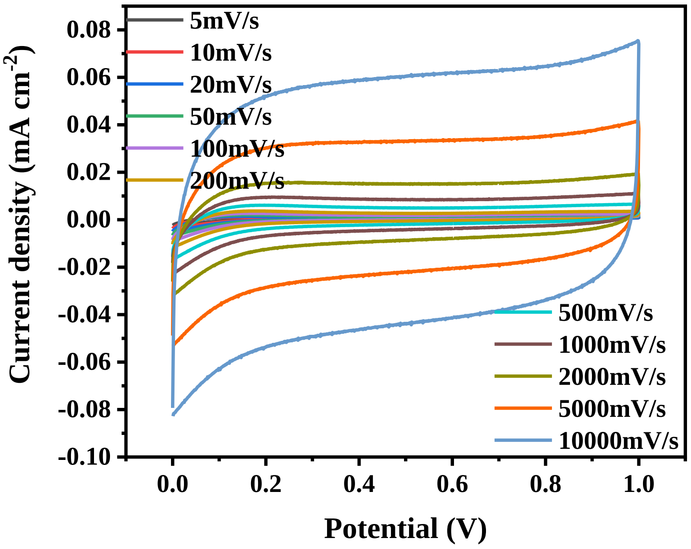

Figure S9. CV curves of TpPB-Fe-based MSC in PVA/H_2SO_4 gel electrolyte at different scan rates.

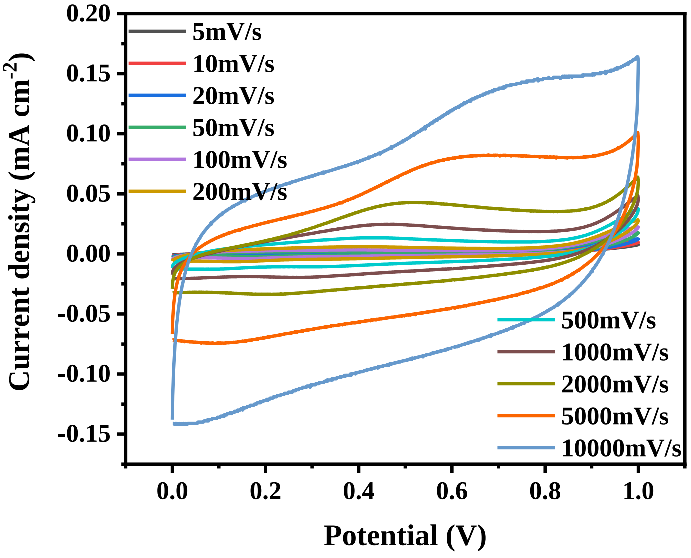

Figure S10. CV curves of TpPB-Fe-based MSC in [EMIM][BF_4] gel electrolyte at different scan rates.

Table S1. Band structure analysis of **TpPB-Fe**.

	UV-vis		CV		
	λ_{abs} (nm)	$E_{bg, optical}$ (eV)	E_{cb} (eV)	E_{vb}[a] (eV)	$E_{bg, CV}$ (eV)
TpPB-Fe	581	1.66	−3.56	-5.22	1.72

[a] the E_{vb} is calculated from the equation of $E_{bg, optical} = E_{cb} - E_{vb}$.

Table S2. The performance of MSCs based on different electrode materials.

Electrode materials	Thickness	electrolyte	Scan rate ($V\ s^{-1}$)	C_V ($F\ cm^{-3}$)	E_V ($mWh\ cm^{-3}$)	reference
**TpPB-Fe**	**300 nm**	**H_2SO_4/ LiCl**	**0.005**	**41.7**	**5.8**	***This work***
			**0.05**	**12.7**	**1.8**	
PiCBA	50 nm	H_2SO_4/ PVA	0.05	34.1	4.7	1
PANI nan-owires	10.1 μm	H_2SO_4/ PVA	0.3 mA cm^{-2}	105	3.9	2
Onion-like carbon	7.0 μm	$TEABF_4$/ PC	1	1.35	1.6	3
		Na_2SO_4 (1 M)	0.04	--	1	
CNT/RGO	6.0 μm	KCl (3 M)	0.01	3.1	0.68	4
CNT/ Graphene	20.0 μm	Na_2SO_4 (1 M)	1.0 A cm^{-3}	1.1	0.16	5
Graphene	15 nm	H_2SO_4/ PVA	0.01	17.9	2.5	6
RGO	--	--	0.02	3.1	0.43	7
graphene	5 μm	$EMIMNTF_2$	0.005	2	1.81	8

Reference:

1. Yang, C.; Schellhammer, K. S.; Ortmann, F.; Sun, S.; Dong, R.; Karakus, M.; Mics, Z.; Loffler, M.; Zhang, F.; Zhuang, X.; Canovas, E.; Cuniberti, G.; Bonn, M.; Feng, X. Coordination Polymer Framework Based On-Chip Micro-Supercapacitors with AC Line-Filtering Performance. *Angew. Chem. Int. Ed.* **2017**, *56*, 3920-3924.
2. Meng, C.; Maeng, J.; John, S. W. M.; Irazoqui, P. P. Ultrasmall Integrated 3D Micro-Supercapacitors Solve Energy Storage for Miniature Devices. *Adv. Energy Mater.* **2014**, *4*, 1301269.
3. Pech, D.; Brunet, M.; Durou, H.; Huang, P.; Mochalin, V.; Gogotsi, Y.; Taberna, P. L.; Simon, P. Ultrahigh-Power Micrometre-Sized Supercapacitors Based on Onion-Like Carbon. *Nat. Nanotechnol.* **2010**, *5*, 651-654.
4. Beidaghi, M.; Wang, C. Micro-Supercapacitors Based on Interdigital Electrodes of Reduced Graphene Oxide and Carbon Nanotube Composites with Ultrahigh Power Handling Performance. *Adv. Funct. Mater.* **2012**, *22*, 4501-4510.
5. Lin, J.; Zhang, C.; Yan, Z.; Zhu, Y.; Peng, Z.; Hauge, R. H.; Natelson, D.; Tour, J. M. 3-Dimensional Graphene Carbon Nanotube Carpet-Based Microsupercapacitors with High Electrochemical Performance *Nano Lett.* **2013**, *13*, 72-78.
6. Wu, Z. S.; Parvez, K.; Feng, X.; Müllen, K. Graphene-Based in-Plane Micro-Supercapacitors with High Power and Energy Densities. *Nat. Commun.* **2013**, *4*, 2487.
7. Gao, W.; Singh, N.; Song, L.; Liu, Z.; Reddy, A. L.; Ci, L.; Vajtai, R.; Zhang, Q.; Wei, B.; Ajayan, P. M. Direct Laser Writing of Micro-Supercapacitors on Hydrated Graphite Oxide Films. *Nat. Nanotechnol.* **2011**, *6*, 496-500.
8. Shi, X.; Pei, S.; Zhou, F.; Ren, W.; Cheng, H.-M.; Wu, Z.-S.; Bao, X. Ultrahigh-Voltage Integrated Micro-Supercapacitors with Designable Shapes and Superior Flexibility. *Energy Environ. Sci.* **2019**, *12*, 1534-1541.
